# CRISPR-Mediated Non-Viral Site-Specific Gene Integration and Expression in T Cells: Protocol and Application for T-Cell Therapy

**DOI:** 10.3390/cancers12061704

**Published:** 2020-06-26

**Authors:** Zelda Odé, Jose Condori, Nicolas Peterson, Sheng Zhou, Giedre Krenciute

**Affiliations:** 1 Department of Bone Marrow Transplantation and Cellular Therapy, St Jude Children’s Research Hospital, Memphis, TN 38105, USA; zelda.ode@gmail.com (Z.O.); jose.condori@stjude.org (J.C.); 2 Graduate School of Biomedical Sciences, St Jude Children’s Research Hospital, Memphis, TN 38105, USA; nicolas.peterson@stjude.org; 3 Experimental Cellular Therapeutics Lab, St Jude Children’s Research Hospital, Memphis, TN 38105, USA; sheng.zhou@stjude.org

**Keywords:** CAR, T cell, non-viral, HDR, CRISPR-Cas9, knock-in, TRAC

## Abstract

T cells engineered with chimeric antigen receptors (CARs) show great promise in the treatment of some cancers. Modifying T cells to express CARs generally relies on T-cell transduction using viral vectors carrying a transgene, resulting in semi-random DNA integration within the T-cell genome. While this approach has proven successful and is used in generating the Food and Drug Administration (FDA, USA) approved B-lymphocyte antigen CD19-specific CAR T cells, it is possible the transgene could integrate into a locus that would lead to malignant transformation of the engineered T cells. In addition, manufacturing viral vectors is time-consuming and expensive. One way to overcome these challenges is site-specific gene integration, which can be achieved through clustered regularly interspaced short palindromic repeat (CRISPR) mediated editing and non-viral DNA, which serves as a template for homology-directed repair (HDR). This non-viral gene editing approach provides a rapid, highly specific, and inexpensive way to engineer T cells. Here, we describe an optimized protocol for the site-specific knock-in of a large transgene in primary human T cells using non-viral double stranded DNA as a repair template. As proof-of-principle, we targeted the T-cell receptor alpha constant (*TRAC*) locus for insertion of a large transgene containing green fluorescence protein (GFP) and interleukin-15 (IL-15). To optimize the knock-in conditions we tested template DNA concentration, homology arm length, cell number, and knock-in efficiency over time. We then applied these established guidelines to target the *TRAC* or interleukin-13 (*IL-13*) locus for the knock-in of synthetic molecules, such as a CAR, bispecific T-cell engager (BiTE), and other transgenes. While integration efficiency depends on the targeted gene locus and selected transgene, this optimized protocol reliably generates the desired insertion at rates upwards of 20%. Thus, it should serve as a good starting point for investigators who are interested in knocking in transgenes into specific loci.

## 1. Introduction

T-cell therapies are widely being explored for the treatment of several clinical indications including multiple types of cancer and autoimmune diseases [1,2]. One therapy involves adoptive transfer of patient-derived T cells that have been engineered to express chimeric antigen receptors (CARs) specific for tumor-associated antigens. CAR engineered T cells are then infused back into the patient and are able to recognize and kill tumor cells expressing targeted antigens on their surface. CAR T-cell therapy has produced outstanding results in CD19 antigen-positive hematological B-cell malignancies resulting in complete response rates of about 83% in relapsed/refractory B-cell acute lymphoblastic leukemia (ALL) patients [3,4,5,6].

Current preclinical and clinical production of CAR T cells relies to a great extent on T-cell transduction using viral vectors (e.g., retrovirus, lentivirus) to deliver transgenes of interest. However, a CAR transgene delivered to a T cell by viral transduction is subjected to random integration into the host DNA. This can lead to unpredictable and variable expression of the transgene. In addition, random integration could lead to a malignant transformation if the transgene is integrated into an oncogenic locus [7,8]. Finally, viral vector production is time-consuming (>6 months), expensive, and poses biosafety challenges [9,10,11,12,13]. 

Genetic engineering approaches that aim to integrate a therapeutic gene into a targeted locus have the potential to solve the problems associated with random integration. Site-specific gene integration can be achieved with the use of gene-editing tools (e.g., CRISPR-Cas9, transcription activator-like effector nucleases (TALEN), zinc finger nucleases (ZFN), meganucleases), which results in DNA double-strand breaks (DSBs) and homology-directed repair (HDR) when a donor DNA template is provided. This approach is known as gene knock-in (KI). In addition, a newly emerging Cas9 base editor technology, which can make gene modifications without DSBs, holds great promise for gene KI in T cells [14]. More and more KI studies are emerging using CRISPR-Cas9 in human T cells to develop clinically useful engineered T cells. In one of the first proof-of-concept studies, a short 12 base pairs (bp) oligonucleotide flanked by 90 bp homology arms was used to create a targeted gene modification at the C-X-C chemokine receptor type 4 (*CXCR4*) locus resulting in 20% knock-in efficiency and creating a targeted gene modification [15]. In another study, applying site-specific DNA integration, programmed cell death protein 1 (PD-1) expression was reduced by 58.6% using a single-stranded DNA oligo (99 bp) carrying a single nucleotide deletion mutation [16].

Recently, Hale et al. reported successful, targeted CD19-CAR integration into the T-cell receptor alpha constant (*TRAC*, 42% of HDR) and C-C chemokine receptor type 5 (*CCR5*; 37% of HDR) loci in T cells using megaTAL nuclease approach and recombinant adeno-associated virus (AAV) for HDR template delivery [17]. Shortly after, Eyquem and colleagues also reported a successful CD19-CAR knock-in to the *TRAC* locus using CRISPR-Cas9 gene editing [18]. This resulted in improved and consistent CAR expression in T cells, decreased baseline (tonic) signaling, and increased anti-tumor activity in vivo when compared to the CAR T cells generated by viral transduction [18]. Similar to the other group, they also used an AAV vector to deliver donor DNA to T cells for HDR-mediated site-specific integration. Such an approach is time consuming, expensive, and labor-intensive because it requires cloning template DNA into the appropriate vector and producing a high titer viral supernatant prior to gene editing. To overcome these obstacles, Roth and colleagues characterized a different method of HDR template delivery. Instead of employing a viral vector, they utilized non-viral double-stranded DNA (dsDNA) as an HDR template, which was generated via conventional PCR amplification [19]. This method results in high-efficiency knock-in and is substantially cheaper and faster than using a viral vector-based delivery. Thus, it has the potential to reduce costs and time for generating targeted gene modifications in human T cells for therapeutic use. 

Here, we describe an optimized step-by-step protocol for a CRISPR-Cas9-mediated knock-in strategy using a dsDNA as a donor DNA template to insert a transgene of interest into a specific region in the T-cell genome. For our knock-in experiments we used non-viral DNA as an HDR template as described in Roth et al. [19]. For the protocol optimization steps, we targeted the *TRAC* locus as the insertion site of our transgene. This genomic region has been used for multiple CRISPR-Cas9-mediated gene integration studies and has been shown to be a stable and safe integration site [17,18,19,20]. Overall, we demonstrated an efficient integration of a large transgene construct into the *TRAC* locus and determined optimal conditions for CRISPR-Cas9-mediated knock-in. We also showed that synthetic gene integration into the *IL-13* locus of T cells can create an inducible system controlled by T-cell activation. 

## 2. Results

### 2.1. Gene Knock-In Using Primary T Cells: Overview

**Figure 1:**
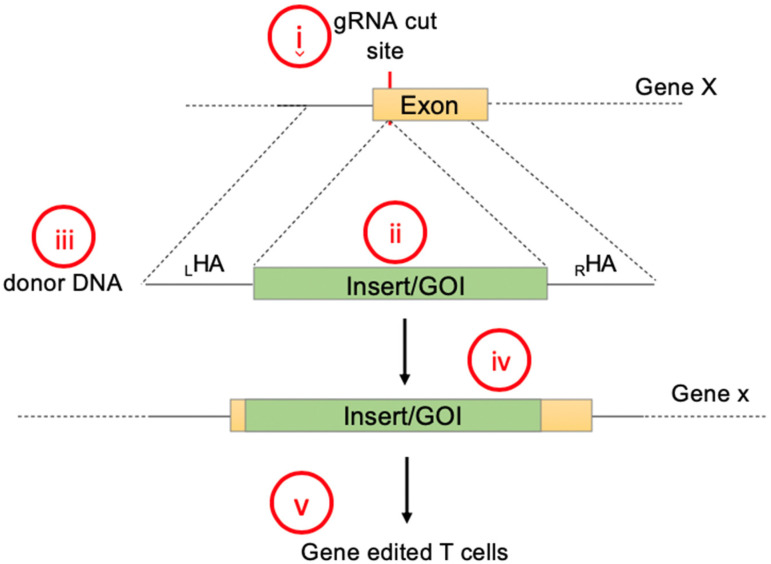
Steps to consider for transgene knock-in using non-viral DNA delivery: (**i**) Target site and guide RNAs, (**ii**) transgene design, (**iii**) donor DNA length, DNA type and delivery method, (**iv**) detection and efficiency of the knock-in, and (**v**) viability and performance of genetically engineered T cell containing the gene of interest.

For protocol establishment, we chose primary human T cells as our target cells because they are clinically relevant. To optimize knock-in conditions we targeted the *TRAC* locus for gene insertion, which has been previously explored for the knock-in of several genes [18,19]. Integration of a promoterless transgene into the *TRAC* locus will disrupt *TRAC* expression. However, the endogenous promoter will continue to drive the expression of the newly inserted synthetic gene. For successful integration of a large transgene, the following elements have to be considered: (1) Target site and guide RNAs (gRNAs), (2) transgene design, (3) donor DNA length, type (single stranded DNA (ssDNA), double-stranded DNA (dsDNA), or plasmid) and delivery, (4) detection and efficiency of the knock-in, and (5) T-cell viability (Figure 1). In our proof-of-concept study, we used two transgenes, IL-15 and mClover3, separated by a 2A sequence. When integrated into the T-cell genome, gene-edited T cells will express mClover3 fluorescent protein [21] and can be readily detected by flow cytometry (green fluorescence protein (GFP) channel). Secretion of IL-15 can be analyzed by ELISA. Importantly, the IL-15 and mClover3 expression cassette is close to the size of a CAR molecule. Hence, our findings can be readily applied for CAR knock-in into human T cells. To optimize the knock-in conditions we evaluated template DNA concentration, cell number, homology arm length, and knock-in efficiency over time, all of which are discussed in detail below. With the optimized protocol, we were able to achieve up to 60% knock-in efficiency and establish guidelines for the gene knock-in in T cells, accelerating the process of T-cell engineering.

### 2.2. Designing Donor DNA

**Figure 2:**
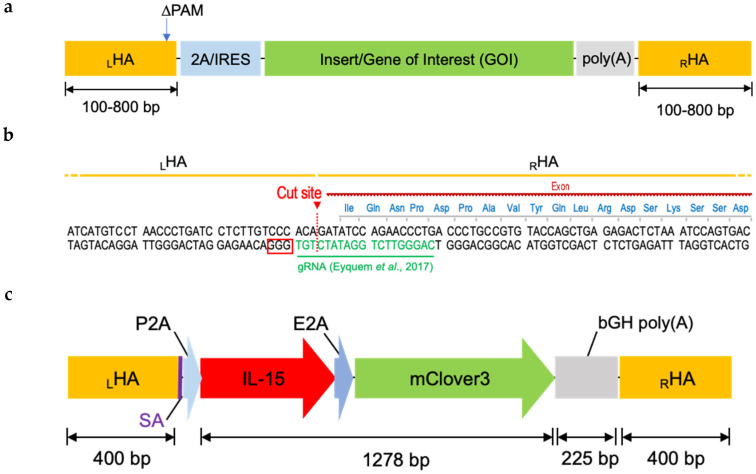
Components of non-viral transgene cassette for expression under endogenous promoter: (**a**) Schematic of non-viral cassette encoding a gene of interest surrounded by a 2A/IRES at the 5′ end for cleavage GOI from endogenous gene, and poly (A) region at the 3′ end for complete termination; _L_HA, left homology arm; _R_HA, right homology arm; ∆PAM, mutated PAM sequence of guide (g) RNA. (**b**) Design of homology arms (HAs) surrounding the cut site of gRNA targeting human *TRAC* locus. PAM sequence is indicated in the red box. (**c**) Scheme of the construct used for the study. It consists of IL-15 and mClover3 for dual transgene expression; SA, splice acceptor; P2A, “self-cleaving” 2A peptide derived from porcine teschovirus-1; E2A, “self-cleaving” 2A peptide derived from equine rhinitis A virus; bGH poly (A), bovine growth hormone polyadenylation signal.

While there are many published studies on gene editing using CRISPR-Cas9-mediated knock-in, there are no universal guidelines on how to design a donor/template DNA for HDR-mediated gene insertion. Donor DNA consists of a gene of interest (GOI) flanked by left and right homology arms (_L_HA and _R_HA), which are sequences homologous to the target locus (Figure 2a). In addition, the donor DNA can also include other elements such as a promoter, enhancer, and 2A self-cleaving peptide or internal ribosome entry site (IRES) sequence at the 5′ and poly(A) signal at the 3′ end. HAs are designed to flank the Cas9 cutting site, with equal length HAs of up to 800 bp per side (Figure 2a,b). Based on these rules, our final donor DNA for the TRAC locus contained the following parts: 400 bp _L_HA, spliced acceptor (SA) [18], P2A (porcine teschovirus-1 2A peptide), IL-15, E2A (equine rhinitis A virus 2A peptide), mClover3, poly(A) (bovine growth hormone polyadenylation signal), and 400 bp _R_HA (Figure 2c). We included a P2A sequence at the 5′ end to separate our transgene from a possible fusion to the endogenous gene, and a poly(A) sequence at the 3′ end for efficient termination as simple STOP codon might not be sufficient. Lastly, we also mutated the protospacer adjacent motif (PAM) sequence in the _L_HA to inhibit the Cas9 enzyme from repeatedly cutting the DNA in this location. The construct was then synthesized by GeneArt and inserted into the pMA plasmid. This plasmid was then used as a template for PCR reaction to amplify dsDNA and to generate donor DNA for HDR-mediated gene knock-in using CRISPR-Cas9.

### 2.3. Donor DNA Amplification, Purification, and Concentration

**Figure 3:**
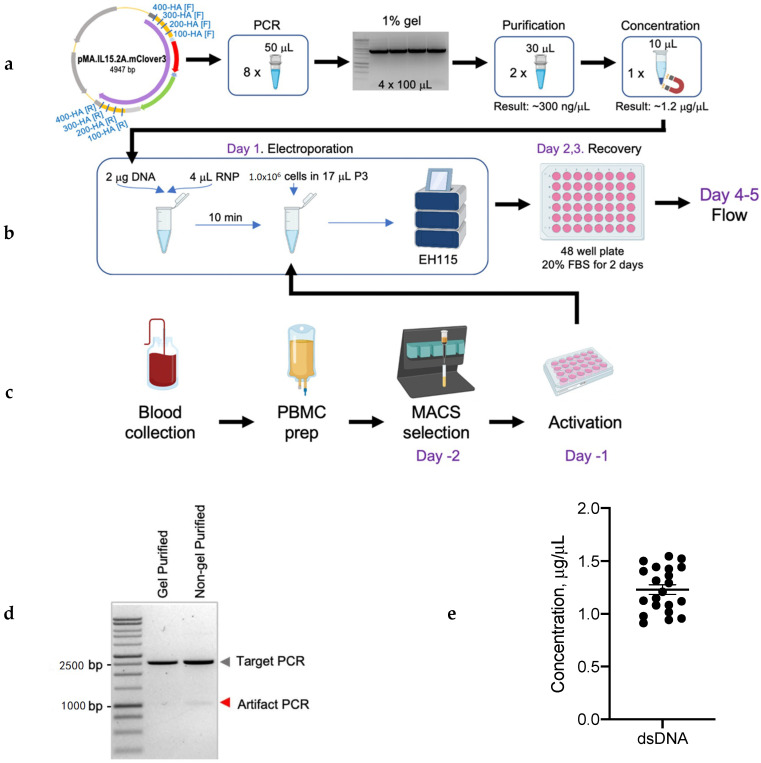
Overview of the steps to generate transgene knock-in in T cell: (**a**) Molecular steps considering vector design, amplification, purification, and concentration DNA. (**b**) Electroporation of RNA ribonucleoproteins (RNPs) and donor template to T cells using Lonza instrument. (**c**) Human T-cell preparation before electroporation. Complete and detailed knock-in protocol can be found in the Supplementary Materials. (**d**) The 1% agarose gel showing PCR amplicons that were gel purified vs. non-gel purified. (**e**) Concentration of dsDNA template after purification and concentration (*n* = 24).

An overview of the donor DNA amplification, purification, and concentration protocol is shown in Figure 3a and detailed knock-in protocol is provided in the Supplementary Materials. Briefly, primers to amplify donor DNA were designed using SnapGene^TM^ for different homology arm lengths (100, 200, 300, and 400 bp) for insertion in the TRAC gene locus. The dsDNA was generated by PCR amplification using CloneAmp HiFi Taq polymerase (Takara Bio, Mountain View, CA, USA), forward and reverse primers, plasmid DNA, and nuclease-free water in a total of 50 µL reaction volume and ran on the ProFlex^TM^ thermocycler (Thermofisher, Waltham, MA, USA). Two PCR reaction products were combined and separated by electrophoresis on 1% agarose gel for DNA size confirmation and gel purification. To generate high amounts of dsDNA, eight PCR reactions were run in total and two of these reactions were combined in one gel slot.

The amplicons with the size that corresponded to the insert with homology arms’ size were excised from the gel (Figure 3d) and gel purified. The products were eluted in total of 60 µL of nuclease-free water. An additional purification step was used to eliminate any toxic leftovers from the gel as well as concentrate dsDNA. For this step we used Agencourt AMPure’s SPRI paramagnetic beads (Beckman Coulter, Pasadena, CA, USA). The final products were eluted in 10–15 µL of nuclease-free water and used in electroporation experiments. This procedure routinely yielded dsDNA at a concentration of 0.9–1.5µg/µL (Figure 3e). At this point, concentrated donor dsDNA was used for the nucleofection (Figure 3b) into activated T cells (Figure 3c), as described in Material and Methods section and the protocol (see Supplementary Materials).

### 2.4. Optimizing Transgene Knock-In in Primary Human T Cells

After we generated donor DNA, we next proceeded with steps to optimize the knock-in efficiency of the IL-15.E2A.mClover3 transgene in primary human T cells. We tested the following parameters: Template dsDNA concentration, cell numbers, homology arm length, and recovery time.

#### 2.4.1. DNA Amount

**Figure 4:**
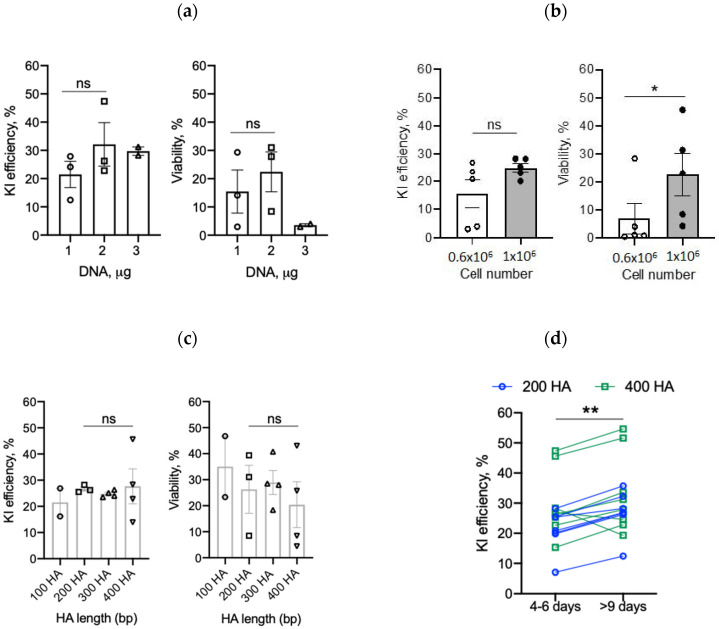
Optimization steps to increase transgene knock-in (KI) efficiency in T cells: (**a**) Three different dsDNA template concentrations were evaluated for the best knock-in efficiency and cell viability (*n* = 3 for 1 and 2 µg, *n* = 2 for 3 µg; two-tailed *t*-test; ns—not significant). (**b**) Two different numbers of T cells were tested for electroporation (*n* = 5, two-tailed paired *t*-test, * *p* = 0.041). (**c**) Four different lengths of homology arms flanking the transgene of interest were evaluated (*n* = 2–4, one-way ANOVA, ns—not significant). (**d**) Knock-in efficiency was tested at early (4–6 days) and late (>9 days) time points post-electroporation (*n* = 15, two-tailed paired *t*-test; ** *p* = 0.0015).

Electroporation of large amounts of plasmid or linear DNA into cells can be toxic, resulting in poor cell viability and high rates of cell death [19,22,23]. To minimize toxicity while maintaining optimal knock-in efficiencies, we first evaluated the effect of different template DNA quantities. We used 1 µg, 2 µg, or 3 µg of IL-15.E2A.mClover3 DNA in 3 µL of nuclease-free water for electroporation together with Cas9: Single-guide (sg) RNA ribonucleoproteins (Cas9 RNPs). Electroporation was performed as shown in Figure 3b and described in the Material and Method section. Briefly, activated T cells were resuspended in P3 electroporation buffer and electroporated with 4 µL of Cas9 RNPs together with 3 µL of donor DNA for HDR. Transgene integration was evaluated 4–6 days later by flow cytometry analysis to determine the percentage of alpha, beta T-cell receptor (TCRαβ)-negative and GFP-positive T cells (referred to as GFP+ cells thereafter, Figure S1a). Electroporation of T cells with TRAC-specific guide RNA with Cas9 RNPs routinely produced around 91% efficient knock-out of the *TRAC* gene as shown in Figure S1b–d. As shown in Figure 4a, 1 µg, 2 µg, or 3 µg of template DNA resulted in an average of 21.5%, 32.1%, or 29.7% knock-in efficiency as judged by GFP+ cells (Figure 4a, left panel) with a cell viability of 15.5%, 22.4%, or 3.6%, respectively (Figure 4a, right panel). Based on this result, we used 2 µg of donor DNA in subsequent experiments.

#### 2.4.2. T-Cell Number

Next, we tested whether increasing the number of T cells per reaction improves T-cell recovery post-electroporation and enhances gene editing efficiency. As shown in Figure 4b, increasing the number of T cells from 0.6 × 10^6^ to 1.0 × 10^6^ per electroporation reaction resulted in a higher KI efficiency (15.6% to 25.0%, Figure 4b); however, it did not reach significance. Importantly, increasing T-cell numbers per reaction significantly (*p* = 0.04) enhanced T-cell survival from 7.0% to 22.6%, respectively (Figure 4b). As a side note, we also found that combining two electroporation reaction vessels into one recovery well (48-well plate) further improved T-cell viability.

#### 2.4.3. Homology Arms and Recovery Time

We next sought to evaluate if longer HA length results in improved knock-in efficiency. To test this, we used our IL-15.E2A.mClover3 transgene flanked by 100, 200, 300, and 400 bp HAs. As shown in Figure 4c, we obtained an average of 21.5, 26.6, 24.8, or 27.7% GFP+ cells, respectively, with no statistically significant difference between groups. In addition, different HA lengths did not affect T-cell viability (Figure 4c, right panel). Taken together, these results indicate that knock-in efficiency of a large transgene is not dependent on HA length tested.

Finally, we evaluated knock-in efficiency at a later time point (>9 days) post-electroporation, which allows T cells to rest, recover, and expand. We found that testing for knock-in efficiency at later time points increased the percentage of GFP+ cells in comparison to early (4–6 days) time points (Figure 4d).

### 2.5. IL-15 Transgene Is Functional

**Figure 5:**
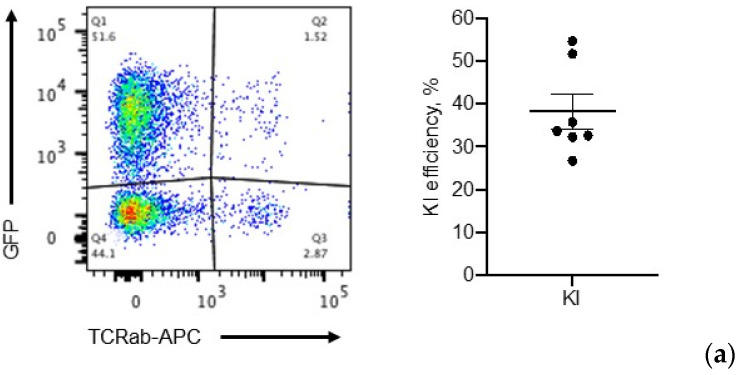

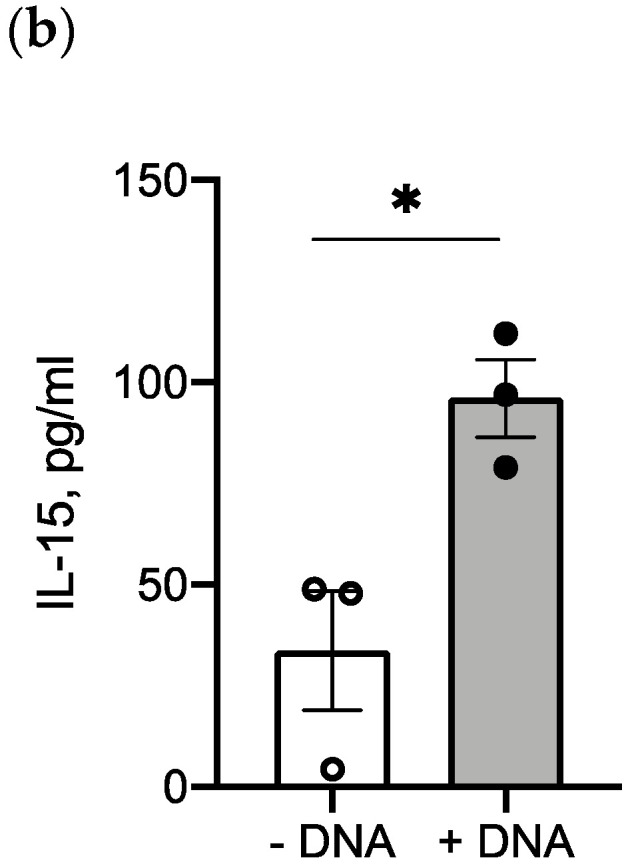
Validating expression of transgenes in gene-edited T cells: (**a**) Representative flow graph of GFP expression in gene-edited T cells 12 days post-electroporation; right panel, overall knock-in efficiency of the transgene after knock-in optimization as determined by flow cytometry of GFP+/TCRαβ- cells (*n* = 7). (**b**) IL-15 production from gene-edited T cells was detected by ELISA 8–10 days post-electroporation (*n* = 3, two-tailed t-test, * *p* = 0.024).

To ensure the robustness of our optimized protocol, three different experimenters performed the knock-in assays, routinely achieving an average of 38.2% KI efficiency (range 26.7–54.6%, Figure 5a). However, up to this point, we evaluated gene editing efficiency based on GFP+ cells. Since IL-15 is also a component of the transgene, we next tested IL-15 secretion from gene-edited T cells via ELISA. Briefly, 8–10 days post-electroporation, 5 × 10^5^ cells were washed with phosphate-buffered saline (PBS), resuspended in 275 µL of media, and plated in 96-well, V-shaped plates. After 24 h, 250 µL of media was collected for IL-15 ELISA. As shown in Figure 5b, gene-edited T cells secreted approximately 2 times as much IL-15 compared to control cells (electroporated without DNA, -DNA).

### 2.6. Application: CAR, BiTE Integration into TRAC Locus and IL-15.E2A.mClover3 Knock-In into IL-13 Locus

Next, we tested if we can knock-in a gene encoding a CAR or bispecific T-cell engager (BiTE) into the *TRAC* locus using our established protocols. As shown in Figure S2a, we were able to knock-in genes encoding a CAR or BiTE into the *TRAC* locus with ~20% efficiency.

**Figure 6:**
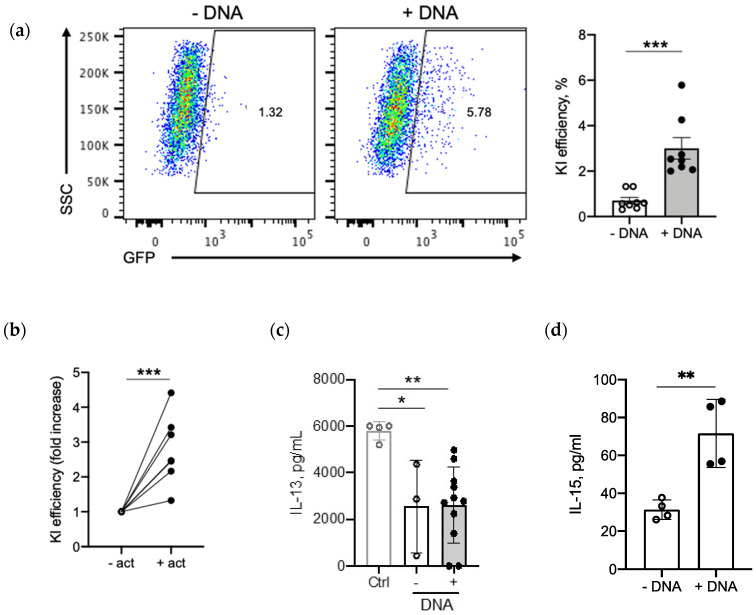
IL-15.E2A.mClover3 knock-in into *IL-13* locus: (**a**) Transgene expression was evaluated by flow cytometry of GFP+ T cells 10 days post-electroporation; left panel, representative flow plots (samples electroporated without template DNA (-DNA) served as controls); right panel, summary graph of left panel (*n* = 8, two-tailed *t*-test; *** *p* = 0.0004). (**b**) Fold increase of knock-in efficiency without (-) or with (+) T-cell activation (*n* = 6, two-tailed *t*-test, *** *p* = 0.0005) (**c**) Knock-out of IL-13 was confirmed by IL-13 secretion; Ctrl, control knock-out; -DNA, knock-in without DNA template; +DNA, knock-in with DNA template (*n* = 3–11, one-way ANOVA, * *p* = 0.036 ** *p* = 0.007). (**d**) IL-15 expression from IL-13-edited (10 days post-electroporation) T cells 24 h post-T-cell activation (*n* = 4, two-tailed *t*-test, ** *p* = 0.005).

To test this protocol further, we asked if we could knock-in our IL-15.E2A.mClover3 transgene into a different gene locus. For that purpose, we picked the *IL-13* locus. We reasoned that, by knocking-in IL-15 into the *IL-13* locus, we can also create an inducible system as IL-13 is highly secreted upon T-cell activation (Figure S2b). We used the same IL-15.E2A.mClover3 transgene flanked by homology arms for the *IL-13* gene locus and performed CRISPR-Cas9-mediated knock-in experiment using our established guidelines. As shown in Figure 6a, we were able to achieve an average of 3% knock-in as judged by flow cytometry of GFP+ cells. Since IL-13 is activation dependent, we next tested if expression of the transgene is affected by T-cell activation. For that, we activated our gene-edited T cells with ImmunoCult™ (Human CD3/CD28/CD2 T Cell Activator, StemCell Technologies, Vancouver, Canada) and quantified GFP+ cells by flow cytometry 24 h later. Indeed, we observed an average of about 3-fold improvement in knock-in efficiency as judged by GFP+ cells in activated samples when compared to non-activated T cells (Figure 6b). In addition, *IL-13* gene knock-out with or without donor DNA led to a significant 2.2-fold decrease in IL-13 secretion (*p* = 0.036 control (ctrl) vs. -DNA and *p* = 0.007 ctrl vs. +DNA), indicating successful *IL-13* gene disruption (Figure 6c). Finally, we tested IL-15 secretion in *IL-13*-edited and -activated T cells. As shown in Figure 6d, we observed a significant increase (average of 2.3-fold) in IL-15 secretion from gene-edited T cells when compared to control T cells. Thus, we can not only knock-in genes of interest into the regions of choice, but also create an inducible system using our optimized protocol.

## 3. Discussion

Here, we demonstrated that primary human T cells can be engineered to express IL-15 and GFP when integrated into the TRAC locus using CRISPR-Cas9 gene editing and non-viral donor DNA as template. In addition, we also showed that we can create an inducible system by inserting IL-15 under the *IL-13* promoter to control IL-15 secretion in a T-cell activation-dependent manner.

The ability to generate T cells expressing a gene-of-interest from a specific locus and/or under a specific promoter opens new avenues for T-cell-based immunotherapies, especially for CAR T-cell-based therapies. Currently, CAR T-cell products are generated mainly by viral transduction, which poses manufacturing challenges as well as safety concerns due to random integration and potential insertional mutagenesis. Here, we provided guidelines on generating template DNA for CRISPR-Cas9-mediated knock-in and performed electroporation to deliver a transgene to the T cells. We believe that this protocol can serve as a primer for designing and performing knock-in experiments. The level of knock-in efficiency achieved here is sufficient for producing a clinically relevant CAR T-cell product. However, for clinical translation and production, the next step would be to scale up the process to edit 1–3 × 10^8^ T cells for future clinical manufacturing. In addition, it is important to mention that in vitro and in vivo studies characterizing and evaluating the long-term safety of CRIPSR-Cas9-mediated knock-in are warranted.

As indicated by our data, knock-in efficiency can widely vary and is dependent on gene-of-choice as well as targeted integration site. As a next step of improving editing efficiencies, several adjustments can be considered. For example, Nguyen et al. recently described two improvements to increase knock-in efficiency [24]. They recommend incorporating truncated Cas9 target sequences at the ends of homology arms. In addition, they also suggested using of polyglutamic acid (PGA), which can stabilize Cas9 RNPs, resulting in an increased editing efficiency while sustaining T-cell viability. However, in our hands, use of PGA improved knock-in efficiency only by 2%. Another modification to consider is the use of single-stranded (ss) DNA instead of dsDNA. The ssDNA triggers a different repair pathway and leads to improved knock-in efficiency and potentially less non-specific integration [25,26]. There are multiple commercially available kits for fast and efficient ssDNA generation and several studies described methods on efficient knock-in generation using ssDNA [19,27].

As we demonstrated successful synthetic gene integration into the *TRAC* locus, we then wanted to apply our established guidelines to integrate *IL-15* into *IL-13* locus to potentially design an inducible system. Our initial detection of GFP+ cells was very low, which was expected, given the fact that IL-13 is only expressed/secreted upon T-cell activation. When we activated gene-edited T cells, we observed an increased percentage of GFP+ cells and increased secretion of IL-15, as assessed by ELISA. However, GFP+ cell number and IL-15 secretion were still relatively low. This, in part, can be explained by incomplete knock-out of *IL-13*. Another possible explanation might be that the *IL-13* promoter might not be strong enough to drive IL-15 expression. To address this, adding a promoter in the DNA template design might solve this issue. However, such approach might render gene expression constitutive; thus, further engineering improvements will have to be considered. Another reason for low efficiency *IL-13* locus editing might be the stability of the locus, which in part is mediated by chromatin accessibility/structure. Recently, Sadelain’s group reported preliminary data on targeting genomic safe harbors (GSH) [28] and their impact on knock-in efficiency and CAR expression over time. Their findings thus far indicate that not every region targeted for site-specific integration can (1) express exogenous genes and (2) sustain expression over time [29]. This might be the case for the *IL-13* locus. To address this, incorporation of insulators or an enhancer might need to be considered when designing donor template.

Although there are already well-established inducible systems regulating transgene expression, such as Syn-Notch and TetON [30,31,32], one hurdle currently limiting their application is the potential for immunogenicity. By knocking in a transgene into a locus that is expressed only under certain conditions (e.g., *IL-13* is only expressed upon T0cell activation), it is possible to use knock-in technology as a non-immunogenic inducible transgene expression system.

We tested multiple variables that can influence T-cell editing efficiency, such as homology arm length, DNA concentration, cell numbers, and time of recovery post-electroporation. We demonstrated that cell number and time of T-cell recovery are important factors for successful large gene integration. Interestingly, our data indicate that there is no difference in knock-in efficiency when using different length of homology arms. This might be due to the size of homology arms we tested. We might observe some differences in editing efficiencies when using longer than 400 bp HAs, such as 800 bp or 1200 bp. While this might be beneficial for improving editing efficiencies, having longer HAs will affect DNA concentration, which then will lead to a lower T-cell viability. However, all of these need further experimental testing.

In summary, we developed a reliable protocol to insert genes into the genome of human T cells using CRISPR-Cas9 gene editing and non-viral donor DNA as template. We believe that it will be useful for investigators as a reference as they embark on using this technology in their laboratories. In addition, we showed that this protocol can be applied for the creation of an inducible expression system.

## 4. Materials and Methods

### 4.1. Generation of DsDNA Donor Template

#### 4.1.1. Construct Design

All constructs for the gene insertion were designed using SnapGene^TM^ and then synthesized by GeneArt/Life Technologies Corporation (ThermoFisher, Waltham, MA, USA), and subcloned into pMA vector as a final product. A detailed description of IL-15.E2A.mClover3 construct is provided in the Results section. For the CAR knock-in experiment described in the Section 2.6 (Figure S1a), we used IL13Rα2-specific CD28.ζ CAR sequence, which has been previously described [33]. The BiTE sequence was as follow: TEM8-specific single-chain variable fragment (scFv) L2 [34,35], a short serine-glycine linker, and CD3-specific scFv [36] followed by 2A sequence and Q8 tag for detection. Both constructs contained HAs for *TRAC* locus.

#### 4.1.2. PCR Amplification

Plasmids were transformed and amplified in DH5α bacterial cells and grown overnight. DNA was extracted by Nucleobond Endotoxin-free Maxiprep (Takara Bio, Mountain View, CA, USA). Primers were designed using SnapGene^TM^ for different homology arm lengths (100, 200, 300, and 400 bp) for insertion in the *TRAC* locus (Table S1). Rules used for primer design were: ±50% CG content, less than 22 bp in length, and melting temperatures below 60 °C. The dsDNA was generated by PCR amplification using CloneAmp HiFi Taq polymerase (Takara Bio, Mountain View, CA, USA), forward and reverse primer (0.5 µM), plasmid DNA (15–20 ng), and nuclease-free water, in a final volume of 50 µL. Reactions were run on the ProFlex^TM^ PCR System (ThermoFisher, Waltham, MA, USA) according to the following program: Initial denaturation at 98 °C for 30 sec, 20 cycles of each 3 steps (denaturation at 98 °C for 10 sec, annealing at +3 °C of lower melting temperature of primer for 15 sec, and extension at 72 °C for variable time based on PCR product size—5 sec/kb), final extension at 72 °C for 3 min. Two PCR reactions combined were run on 1% agarose gel for band size confirmation. The BenchTop 1kb DNA Ladder (Promega, Madison, WI, USA) was used in all experiments. To generate highly concentrated dsDNA, 8 PCR reactions were run in total and 2 of these reactions were combined in one gel slot.

#### 4.1.3. DNA Purification and Concentration

The amplicons with the size-corresponding transgene DNA size were excised from the gel and purified using the NucleoSpin^®^ Gel and PCR clean up kit (Takara Bio, Mountain View, CA, USA). The products were eluted in 60 µL of nuclease-free water and used for further concentration. An additional purification step was used to eliminate any toxic leftovers from the gel. For this step, we used Agencourt AMPure’s SPRI paramagnetic bead technology (Beckman Coulter, Pasadena, CA, USA). The final products were eluted in 10–15 µL of nuclease-free water (to get a final concentration of ~1–1.5 µg/µL) and used in electroporation experiments.

### 4.2. Generation of Knock-In T Cells

#### 4.2.1. Primary Human T-Cell Culture

Human peripheral blood mononuclear cells (PBMCs) were obtained from whole blood of healthy donors under the Institutional Review Board (IRB)-approved protocols at St. Jude Children’s Research Hospital. To generate gene-edited T cells, we isolated PBMCs by Lymphoprep (Abbott Laboratories, Chicago, IL, USA) gradient centrifugation. CD4+/CD8+ T cells were then enriched from the PBMCs using human anti-CD4- and anti-CD8-specific MicroBeads kit (Miltenyi Biotec, Bergisch Gladbach, Germany), according to the manufacturer’s protocol. Enriched T cells were plated in a 24-well, non-tissue culture-treated plate at 0.5 × 10^6^ cells/mL in 2 mL T-cell media (PMI (GE Healthcare Life Sciences, Marlborough, MA, USA) containing 10% fetal bovine serum (FBS) (GE Healthcare Life Sciences, Marlborough, MA, USA), and 1% GlutaMAX-I (Invitrogen, Carlsbad, CA, USA). The next day, selected T cells were stimulated with 25 µL Human T-Activator CD3- and CD28-specific Dynabeads (ThermoFisher, Waltham, MA, USA) and grown in the T-cell media supplemented with recombinant human IL-7 and IL-15 cytokines (IL-7: 10 ng/mL, IL-15: 5 ng/mL, PeproTech Cranbury, NJ, USA).

#### 4.2.2. Primary Human T-Cell Electroporation

Two days after T-cell activation, cells were electroporated to enable site-specific knock-in using Cas9 RNPs. All electroporation experiments were performed on the 4D-Nucleofector^TM^ System X Unit (Lonza, Basel, Switzerland) using the EH-115 program. RNPs were pre-complexed at a sgRNA:Cas9 ratio of 4.5:1, prepared by adding 3 µL of 60 µM sgRNA (Synthego Menlo Park, CA, USA) to 1 µL of 40 µM Cas 9 (QB3 Macrolab, University of California, Berkeley, CA, USA), and incubated for 10 min at room temperature (RT). Complexed RNPs were used right away or frozen for later use. Sequences for all sgRNAs can be found in Table S2. T cells (0.6 × 10^6^ or 1.0 × 10^6^) were re-suspended in 17 µL P3 buffer including supplement 1 (Lonza). Subsequently, 4 µL of RNP complex was added together with the dsDNA template donor (2 µg/3 µL unless stated otherwise) and incubated for 10 min at room temp. The RNP and dsDNA mix were added to the cell mixture and 23 µL was added to the transfection vessel and electroporated. After electroporation, 80 µL of recovery media (RPMI (GE Healthcare Life Sciences, Marlborough, MA, USA) including 20% FBS (GE Healthcare Life Sciences, Marlborough, MA, USA), 1% GlutaMAX-I (Invitrogen, Carlsbad, CA, USA), IL-7 at 10 ng/mL, and IL-15 at 5 ng/mL) was added to the electroporation vessel. The cells were rested for 30 min at 37 °C and 5% CO_2_ before being transferred into a 48-well, tissue culture plate with 650 µL of recovery media. Two to three days after electroporation, the FBS concentration was reduced to 10% in the T-cell culture media with cytokines. T cells were split every 3–4 days and fresh IL-7 and IL-15 cytokines were added.

### 4.3. Flow Cytometry

Cells were examined by flow cytometry 4 to 6 or >9 days after electroporation to determine the knock-out and knock-in efficiencies of the desired gene constructs. All flow cytometry experiments were performed on the FACSCanto™ instruments (BD Bioscience, San Jose, CA, USA). FACSDiva (BD Biosciences, San Jose, CA, USA) and FlowJo v.10 (FlowJo, Ashland, OR, USA) were used for analyzing the acquired immunofluorescence data. For surface staining, samples were washed with and stained in PBS (Lonza) with 1% FBS (GE Healthcare Life Sciences, Marlborough, MA, USA). For all experiments, matched isotypes or known negatives (e.g., non-edited or knock-out only T cells) served as gating controls.

Live cells’ populations were evaluated based on SSC-A over FSC-A gating [37] or using LIVE/DEAD Fixable Aqua Dead Cell Stain Kit (Invitrogen, Carlsbad, CA, USA) as a viability dye. TRAC expression was determined by using a mouse anti-human TCRαβ-APC or TCRαβ-PE antibody (BD Biosciences, San Jose, CA, USA). The mClover3, which is protein with a higher fluorescence signal of a jellyfish GFP, positive cells (referred to as GFP+ cell in the text) were detected in the GFP channel [21]. Detection of IL13Rα2-CAR was achieved with recombinant human IL13Rα2 protein conjugated to PE (Creative BioMart, Shirley, NY, USA). Expression of Q8 was detected using anti-human CD34 (Qbend 10) APC antibody (R&D Systems, Minneapolis, MN, USA).

### 4.4. Targeted Deep Sequencing

The hTRAC-specific amplicons were generated using gene-specific primers with partial Illumina adapter overhangs (hTRAC.F – 5′-AGTGTAATACCTTGCAGCACCAGAGC-3′ and hTRAC.R – 5′-TTGCTCCAGGCCACAGCACTGTTGC-3′, overhangs not shown) as previously described [38]. Briefly, hTRAC-specific amplicons were generated, indexed, and pooled with other targeted amplicons for other loci. Additionally, 10% PhiX Sequencing Control V3 (Illumina, San Diego, CA, USA) was added to the pooled amplicon library prior to running the sample on an Illumina Miseq sequencer to generate paired 2 × 250 reads. Samples were demultiplexed using the index sequences, fastq files were generated, and Next-Generation Sequencing (NGS) analysis was performed using CRIS.py [39].

### 4.5. Analysis of IL-15 and IL-13 Production

IL-15 production was measured using a Human IL-15 Quantikine ELISA Kit (R&D Systems, Minneapolis, MN, USA) according to the manufacturer’s protocol. IL-13 production was analyzed using Human IL-13 Quantikine ELISA Kit (R&D Systems, Minneapolis, MN, USA). For measuring IL-15 secretion upon T-cell activation, 5.0 × 10^5^ T cells were washed with PBS, resuspended in 275 µL of T-cell media (RPMI, 10% FBS, and 1% GlutaMAX) without cytokines and plated in 96-well, V-shaped plates. Cells were then activated with Immunocult™ Human CD3/CD28/CD2 T Cell Activator (StemCell Technologies, Vancouver, Canada) following the manufacturer’s protocol and incubated at 37 °C. After 24 h, 250 µL of media was collected for IL-15 ELISA from the wells and stored at −80 °C.

### 4.6. Statistical Analysis

All statistical analyses were performed in GraphPad PRISM 8 (GraphPad Software, San Diego, CA, USA). All experiments were performed at least in duplicates. For comparison between two groups, two-tailed *t*-test was used. For comparisons of three or more groups, values were log transformed as needed and analyzed by ANOVA. *p* values <0.05 were considered statistically significant.

## 5. Conclusions

CRISPR-Cas9 knock-in approaches allow for efficient and fast site-specific gene integration in primary human T cells when donor DNA is provided in non-viral form. This allows for efficient generation of gene-edited T cells expressing multiple therapeutically relevant genes. Here we provided guidelines to streamline donor DNA design and maximize editing efficiency for CRISPR-Cas9 gene editing (Table 1).

## Figures and Tables

**Table 1:** Electroporation checklist for large gene knock-in in human T cells.

sgRNA	3 µL [60 µM]
**Cas9**	1 µL [40 µM]
**sgRNA:Cas9 (molar ratio)**	4.5:1
**RNP incubation**	10 min, RT
**RNP volume**	4 µL
**Cells**	1 × 10^6^/17 µL
**Electroporation solution**	P3 + S1 supplement (Lonza)
**Template**	2 µg dsDNA in 3 µL
**Homology arm length**	200–400 bp
**Vol for electroporation**	23 µL
**Format/program Neon**	Strip/EH-115
**Incubation after electroporation**	30 min at 37 °C (in 80 µL of RPMI+20%FBS+IL7/15)
**Transfer**	48-well plate (650 µL of RPMI+20%FBS+IL7/15)
**Reactions per well after electroporation**	2

## Supplementary Materials

The following are available online at https://www.mdpi.com/2072-6694/12/6/1704/s1, Figure S1. IL-15.E2A.mClover3 transgene integration into *TRAC* locus: (a) Gating strategy for detection of transgene expression in T cells by flow cytometry. (b) Knock-out efficiency of TCR in T cells without (-) or with (+) dsDNA template as measured by flow cytometry (*n* = 5–12). (c) Editing of *TRAC* locus with gRNA (Eyquem et al., 2017) as determined by targeted NGS, *n* = 3. (d) Summary of the most frequent indels by deep sequencing after editing of TCR locus in T cells (sample representative from *n* = 3). The asterisk indicates an unedited allele. Figure S2. Applications of site-specific transgene integration using CRISPR-Cas9: (a) Successful knock-in of a CAR and a BiTE into the *TRAC* locus was confirmed by flow cytometry. For details on CAR and BiTE sequences, see Section 4.1.1. (b) IL-13 production is increased upon T-cell activation (-act, non-activated; +act, activated) (*n* = 3). Knock-In Protocol: Table S1. Primer sequence for dsDNA template. Table S2. SgRNA sequences for TRC locus. Complete and detailed knock-in protocol can be found in Supplementary Materials.
