# Supplementary material for: CRISPR-Mediated Non-Viral Site-Specific Gene Integration and Expression in T Cells: Protocol and Application for T-Cell Therapy
Source: Cancers (Basel). 2020 Jun 26;12(6):1704. doi: 10.3390/cancers12061704 (PMC7352666; doi:10.3390/cancers12061704)
Supplement: Supplementary file 1 [file cancers-12-01704-s001.pdf]

Supplementary Materials

CRISPR-Mediated Non-Viral Site-Specific Gene Integration and Expression in T Cells: Protocol and Application for T-Cell Therapy

Zelda Odé, Jose Condori, Nicolas Peterson, Sheng Zhou and Giedre Krenciute

Supplementary Material: Knock-in Protocol

Biologicals

Healthy donor human blood

Reagents

Lymphoprep (StemCell Technologies; cat. #07801)
HyClone Fetal Bovine Serum (FBS) (Fisher Scientific, SH30071.03, Lot AD20618263)
RPMI-1640 (without L-glutamine; GE Healthcare Life Sciences; cat. #SH30096.01)
GlutaMAX™ (Gibco; cat. #35050061)
PBS (without calcium, magnesium; GE Healthcare Life Sciences; cat. #SH30256.01)
Ethanol, Molecular biology grade (Fisher, cat. #BP2818-100)
Trypan Blue solution (Sigma; cat. #T8154)
IL-7 (Miltenyi Biotec; cat. #130-095-363 or PeproTech; cat. #200-07)
IL-15 (Miltenyi Biotec; cat. #130-095-765 or PeproTech; cat #200-15)
CD4 MicroBeads, human (Miltenyi Biotec; cat. #130-045-101)
CD8 MicroBeads, human (Miltenyi Biotec; cat. #130-045-201)
MACS BSA stock solution (Miltenyi Biotec; cat. #130-091-376)
0.5M EDTA pH8.0 (Life Technology; cat. #15575-038)
P3 Primary Cell 4D-Nucleofector X Kit S (Lonza; cat. #V4XP-3032)
sgRNA targeting TRAC exon1 (Synthego)
Nuclease-free Water (provided with guides, Synthego)
1xTE Buffer pH8.0 (provided with guides, Synthego)
Cas9 enzyme (MacroLab, Berkeley)
Dynabeads Human T-Activator CD3/CD28 (Thermofisher; cat. #11131D)
T Cell TransAct (Miltenyi Biotec; cat. # 130-111-160)
HyClone HyPure Water, Molecular Biology Grade (GE Healthcare Life Sciences; cat. #SH30538.01)
ImmunoCult™ Human CD3/CD28/CD2 T Cell Activator (StemCell Technologies; cat. #10970)
NucleoSpin Gel and PCR Clean-up Kit (Takara; cat. #740609.50)
Agencourt AMPure XP (Beckman Coulter; cat. #A63880)
CloneAmp HiFi PCR Premix (Takara; cat. #639298)
Benchtop 1kb DNA ladder (Promega; cat. #G7541)
Gel Loading Dye, Purple 6X (New England BioLabs; cat. #B7024S)
TopVision Agarose Tablets (Thermo Scientific, cat. #R2801)
UltrPure TAE Buffer 10X (Invitrogen, cat. #15558042)
Mouse anti-human TCRαβ-APC (Clone T10B9.1A-31; BD Biosciences; cat. #563826)
Mouse Isotype Control IgM-APC, κ (Clone G155-228; BD Biosciences; cat. #550883)
P3 Primary Cell 4D-Nucleofector™ X Kit S (Lonza; cat. #V4XP-3032)
Plasmids: custom made from GeneArt

Plasticware and Equipment

24 well tissue culture treated plates (Corning; cat #353047)
48 well tissue culture treated plates (Corning; cat. #353078)
PCR tubes (Applied BiosystemsTM; cat #A30588)
1.5 mL Eppendorf tubes USA Scientific, cat. #1615-5510)
15 mL Falcon tubes (Fisher Scientific; cat. #14-959-53A)
Flowmi cell strainer (Belart, cat. #H13680-0040)
Falcon Round-Bottom Polystyrene Tubes (Corning, cat. #352057)
MidiMACS Separator (Miltenyi Biotec; cat. #130-042-302)
MACS LS columns (Miltenyi Biotec; cat. #130-042-401)
MACS MultiStand (Miltenyi Biotec; cat. #130-042-303)
MiniMACS Separator (Miltenyi Biotec; cat. #130-042-102)
DynaMag™ – Spin Magnet (Thermo Fisher; cat. #12320D)
BD FACSCanto II (BD Biosciences)
Applied Biosystems ProFlex PCR System (Thermo Fisher; cat. #4484073)
NanoDrop™ (Thermo Fisher; cat. #ND-ONEC-W)
4D-Nucleofector™ X Unit (Lonza; cat. #AAF-1002X)
4D-Nucleofector™ Core Unit (Lonza; cat. #AAF-1002B)
Owl™ EasyCast B2 Mini Gel Electrophoresis System (Thermo Scientifc™; cat. #B2-BP)
Countess II FL automated cell counter (Invitrogen, cat. #AMQAF1000)
Gel Doc EZ System (Bio-Rad; cat. #1708270)

Software

SnapGene v4 (GSL Biotech)
FlowJo v9 (BD Biosciences)
BD FACSDiva (BD Biosciences)
PRISM v8.4. (GraphPad)

Donor DNA Design

Suggestions for donor DNA design are illustrated in Figure 2. It is recommended to design the homology arms right next to the cut site of the sgRNA of the targeted gene locus. Based on our protocol optimization we recommend using homology arms of 400 bp for longer insert sizes. A 2A or IRES sequence should be implemented in front of the transgene to ensure proper separation of the product from the native gene product. When two genes are cloned together in one construct, it is recommended to add a 2A sequence in between those genes to avoid fusion genes during translation. At the end of the transgene, before the 5′ end of the right homology arm, a poly A sequence is beneficial for appropriate gene termination. Lastly, mutating the PAM sequence in the construct inhibits the Cas9 enzyme from repeatedly cutting the DNA in this location.

PBMC Preparation

For our studies we isolated PBMCs by Lymphoprep (Abbott Laboratories, Chicago, IL, USA) gradient centrifugation. Generally, any PBMC isolation method is appropriate for this protocol as long as it produces healthy and viable PBMCs. While we used cryopreserved PBMCs, using fresh PBMCs would result in a higher viability of T cells.

T Cell Enrichment

This part of the protocol is a modified version of the MidiMACS kit protocol.

1. Thaw a vial of PBMCs (prepared as described above) in a 37 °C water bath. Wash the thawed PBMCs with T cell media (RPMI-1640 media including 10% FBS and 1% GlutaMAX-I). Resuspend the cell-pellet with MACS buffer (500 mL PBS, 25 mL MACS BSA stock solution, and 2 mL 0.5 M EDTA pH 8.0).
2. Count the cells and spin down at 400g for 5 minutes at room temperature. Aspirate the MACS buffer and add 250 µL of MACS buffer to the cell pellet.
3. Add 20 µL of CD4 and 20 µL of CD8 MicroBeads per 10^7 cells. Ensure proper mixing and incubate for 15 minutes at 4 °C.
4. After incubating, wash the cells with 2 mL of MACS Buffer and spin down at 400 g for 5 minutes at room temperatures. During the wash step, place the MidiMACS Separator on the MACS MultiStand and a MACS LS column in the MidiMACS Separator and equilibrate the column with 3 mL MACS buffer.
5. After washing, aspirate the supernatant and resuspend the stained cells with 500 µL of MACS Buffer. Run the cell suspension through a 40 µM cell strainer to remove dead cell clumps and apply it onto the column.
6. Wash the column with 3 mL of MACS buffer. Repeat this step for a total of 3 washes.
7. Remove the column from the MidiMACS Separator and place on a 15 mL collection tube. Add 5 mL of MACS buffer and insert the syringe plunger (comes with the MACS LS column) to flush the column and release the cells into the collection tube. Spin at 400 g for 5 minutes at room temperature.
8. Add 5 mL of T cell media and count the cells. Plate 10^6 selected T cells in 2 mL T cell media in one well of a 24 well tissue culture treated plate.
9. Rest the plated T cells overnight at 37 °C and 5% CO_2.

T Cell Activation

Here we tested 3 approaches to activate T cells: plate bound CD3/CD28, Dynabeads and Transact. While we did not observe any differences between Dynabeads and Transact, plate bound CD3/CD28 resulted in lower knock-in efficiencies as well as T cell viability. Thus, below we describe two methods for T cell activation.

Dynabeads

1. The next day, transfer Dynabeads Human T-Activator CD3/CD28 (25 μL per well of plated T cells) to a 1.5 mL collection tube.
2. Add 1mL MACS buffer to the tube with Dynabeads and place the tube on the back side of a MiniMACS Separator. With the tube pressed against the magnet, remove the MACS buffer.
3. Re-suspend the beads in T cell media in the same volume as the volume of Dynabeads in step 1.
4. Add 25 μL Dynabeads to each well of plated T cells. Add IL-7 at 10 ng/mL and IL-15 at 5 ng/mL.
5. Rest the activated T cells for two days at 37 °C and 5% CO_2 before electroporation.

TransAct Media

1. The next day, add 28.5 μL T cell TransAct to each 24 well tissue plate with T cells. Add IL-7 at 10 ng/mL and IL-15 at 5 ng/mL.
2. Rest the activated T cells for two days at 37 °C and 5% CO_2 before electroporation.

HDR Template Preparation

1. Design primers to amplify HDR template. (Note: we recommend designing primers 400 bp away from the Cas9 cut site in order to create 400 bp long homology arms)
2. Prepare the PCR mix as listed in Table 1 and keep op ice (8 tubes/reactions are recommended).

Table 1. PCR mix per reaction.

Volume	Reagent
25 μL	CloneAmp HiFi PCR Premix
2.5 μL	Forward primer (10 uM)
2.5 μL	Reverse primer (10 uM)
1 μL	Plasmid DNA (15–20 ng)
19 μL	Nuclease-free water
**50 μL**	**Total**

3. Run the PCR reaction on the Applied Biosystems ProFlex or similar PCR System according to the program in Table 2.

Table 2. PCR program (polymerase dependent protocol).

Step	Temperature	Duration	Cycles
Denature	98 °C	30 seconds	1
Denature	98 °C	10 seconds	20
Annealing	+3 °C of lowest primer Tm	15 seconds	
Elongation	72 °C	5 seconds/kb	
Final elongation	72 °C	3 minutes	1
	4 °C	∞	1

Prepare 1% agarose gel. Stain PCR reactions with loading dye 1X and load 2 PCR reactions in one gel well (100 μL PCR reaction total per well). Load Benchtop or similar DNA ladder and run the gel.

Image the gel and cut out bands with the appropriate band size.

Perform gel extraction with NucleoSpin Gel and PCR Cleanup kit according manufacturer's protocol. Elute all gel pieces in 2 × 30 μL nuclease-free water total (use 2 columns and one collection tube).

Concentrate DNA further with Agencourt AMPure XP beads according to manufacturer's protocol. Elute the final solution from step 5.7. in 10 μL nuclease-free water (recommended final concentration 1–2 μg/μL).

Measure the final DNA concentration on NanoDrop.

Electroporation

Prepare RNP complexes using a 4.5:1 sgRNA:Cas9 molar ratio (carried out in a RNA-free environment):

sgRNA working stock preparation: spin down sgRNA tube and add 10 μL 1× TE to make a 150 μM stock. Dilute in 15 μL RNase-free water to generate a 60 μM working stock solution. Transfer 3 μL TRAC exon 1 sgRNA from the 60 μM stock to a PCR tube.

Add 1 μL cas9 from 40 μM stock to PCR tube containing the sgRNA and incubate for 10 minutes at room temperature (store at −20 °C until use).

Set up recovery plates for the T cells after electroporation (Note: 2 electroporation reactions of 1 × 10^6 cells each combined in one recovery well is recommended for optimal viability and knock-in): 48 well tissue culture treated plate including 550 μL recovery media (RPMI-1640 media including 20% FBS, 1% GlutaMAX-I, IL-7 at 10 ng/mL and IL-15 at 5 ng/mL). The total final volume per well should be 750 μL. (Note: if knock-out efficiency is low, use 0.6 × 10^6 cells per electroporation reaction).

Prepare P3 Primary Cell Nucleofector Solution (17 μL total volume per electroporation reaction): 13.94 μL P3 Primary Cell Nucleofector Solution and 3.06 μL Supplement 1.

Collect the T cells in a 1.5 mL collection tube and remove the Dynabeads Human T-Activator CD3/CD28 from T cells by pressing the tube against a MiniMACS Separator. Take out the cell solution without the Dynabeads. (Note: if T cells were activated with T cell TransAct, transfer the activated T cells to a collection tube).

Count the T cells and take 10^6 cells per electroporation reaction (do not spin cells prior to counting). Spin down the T cells at 200 g for 10 minutes at room temperature.

Remove all the media from the cell pellet and re-suspend the cell pellet in P3 Primary Cell Nucleofector Solution (1 × 10^6 T cells per 17 μL P3 Primary Cell Nucleofector Solution)

Add 2 μg of HDR template (obtained in step 4.9) in no more than 3 μL nuclease-free water together with 4 μl of RNP (obtained in step 5.1) in a new collection tube. Incubate for 10 minutes at room temperature. (Note: always prepare the following controls: No HDR template + RNP; no HDR template, no RNP; and HDR template, no RNP).

Add 17 μl of the T cells in P3 Primary Cell Nucleofector Solution to the tube with HDR template and RNP (and to the control tubes).

Transfer 23 μl of T cells with HDR template and RNP (and of the controls) to one well of the Nucleocuvette Strip and nucleofect the cells using program: EH-115.

After nucleofection, add 80 μl of recovery media to the T cells in the Nucleocuvette Strip (as per manufacturer's suggestions, do not add the media directly into the well but slowly pipet at the rim of the well).

Let T cells to recover at 37 °C and 5% CO_2 for 30 minutes. Then transfer the cells to the recovery plate prepared in step 5.2 (2 electroporation reactions into 1 well of the recovery plate).

T Cell Expansion

Incubate the electroporated T cells at 37 ° C and 5% CO_2. Split the cells when the media yellows and the cells are at high density (this can take a few days as the cell will grow slowly right after electroporation).

Dilute the media to 10% FBS using serum-free RPMI-1640 containing 1% GlutaMAX-I only, 2–3 days after electroporation.

Add IL-7 at 10 ng/mL and IL-15 at 5 ng/mL every 2–3 days.

Determine Knock-in

Determine knock-in efficiency 8–10 days post-electroporation. Detection method is based on the transgene. Detection should be done at a protein level using flow cytometry, ELISA or WB to confirm that the protein is expressed. In addition, targeted next-generation sequencing (NGS) is highly recommended to determine editing efficiency.

Table 1. Primer sequences.

Primer	Primer sequence (5′–3′)	Tm (°C)
TRAC 100 HA	F: CTTGTCCATCACTGGCATCTG	55.9
	R: CGGTGAATAGGCAGACAGAC	55.2
TRAC 200 HA	F: GCCAAGATTGATAGCTTGTGC	54.5
	R: GTCAGATTTGTTGCTCCAGGC	56.6
TRAC 300 HA	F: GCAGTATTATTAAGTAGCCCTG	50.7
	R: CGAAGGCACCAAAGCTG	54.3
TRAC 400 HA	F: CAGTTTGCTTTGCTGGGCCTT	59.1
	R: GGCAATGGATAAGGCCGA	55.2

Table S2. sgRNA sequences.

sgRNA	Sequence (5′–3′)
TRAC	CCCACAGATATCCAGAACCCTG (Eyquem, J., et al, Nature, 2017)
IL-13	g12 - GAGGAGCGGATGCATAGGCTNGG
	g10 – GGATTGAGGAGCGGATGCATNGG

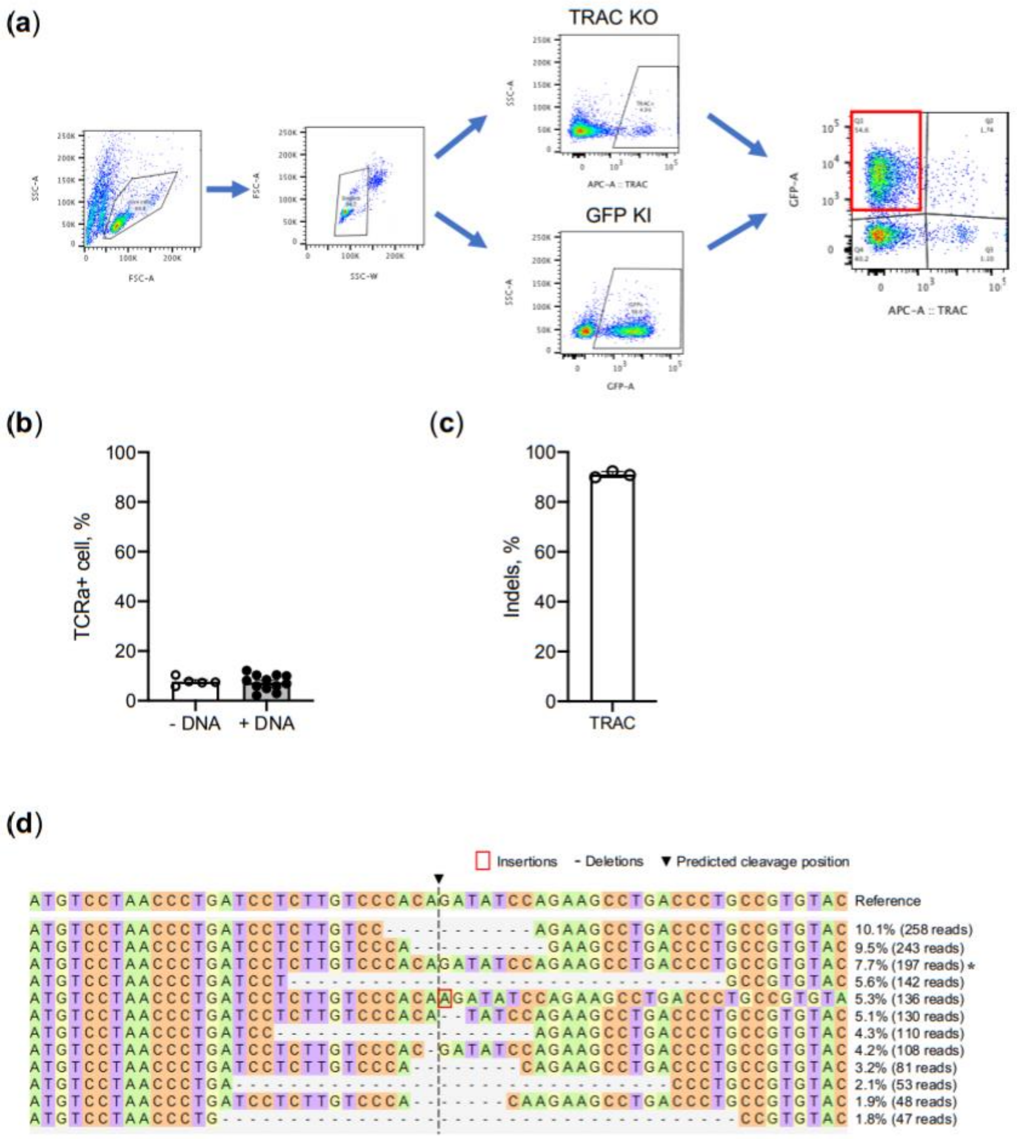

Figure S1. IL-15.E2A.mClover3 transgene integration into *TRAC* locus: (a) Gating strategy for detection of transgene expression in T cells by flow cytometry. (b) Knock-out efficiency of TCR in T cells without (−) or with (+) dsDNA template as measured by flow cytometry (n = 5–12). (c) Editing of *TRAC* locus with gRNA (Eyquem et al., 2017) as determined by targeted NGS, n = 3. (d) Summary of the most frequent indels by deep sequencing after editing of TCR locus in T cells (sample representative from n = 3). The asterisk indicates an unedited allele.

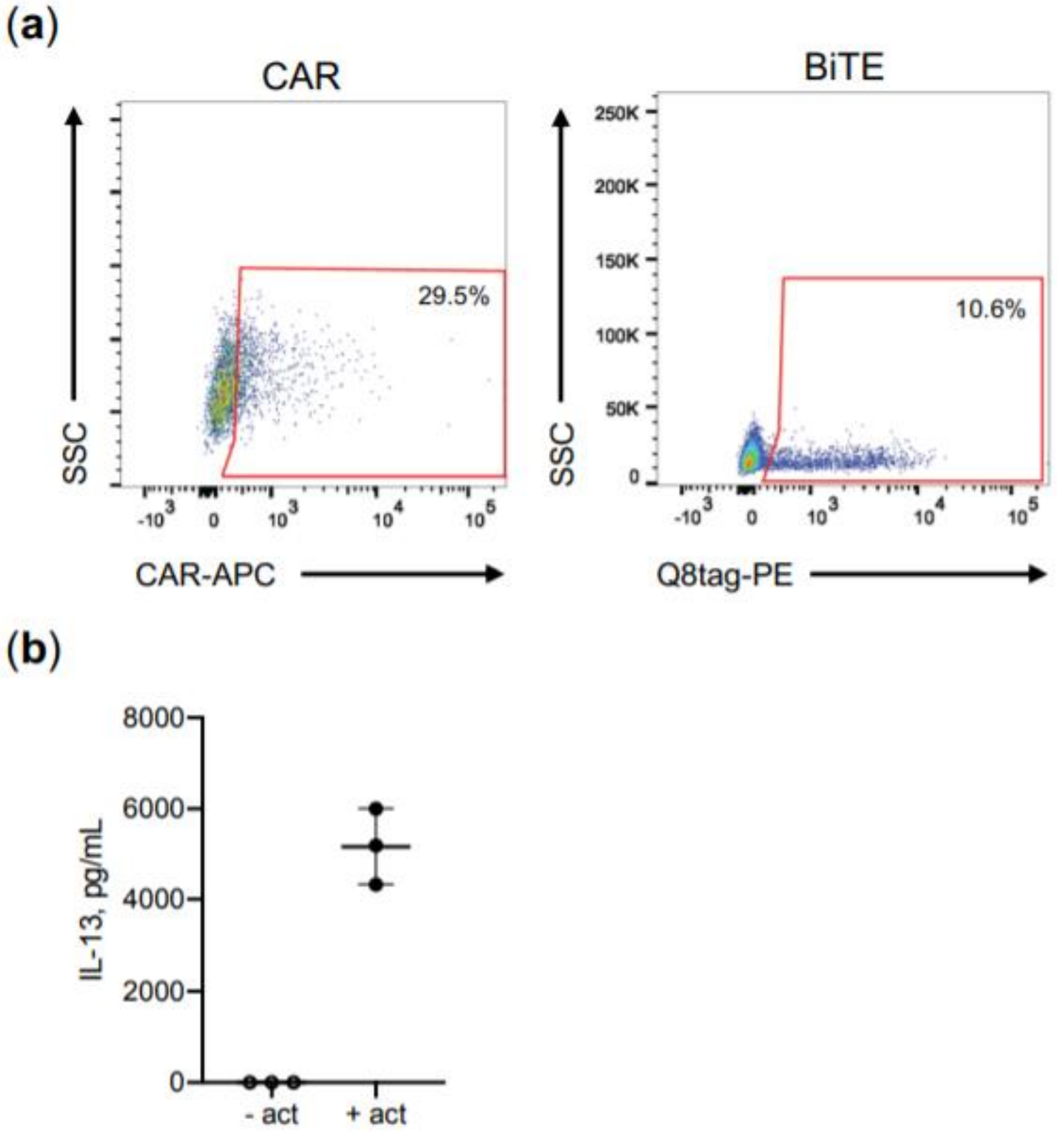

Figure S2. Applications of site-specific transgene integration using CRISPR-Cas9: (a) Successful knock-in of a CAR and a BiTE into the *TRAC* locus was confirmed by flow cytometry. For details on CAR and BiTE sequences, see Section 4.1.1. (b) IL-13 production is increased upon T-cell activation (−act, non-activated; +act, activated) (n = 3).

© 2020 by the authors. Licensee MDPI, Basel, Switzerland. This article is an open access article distributed under the terms and conditions of the Creative Commons Attribution (CC BY) license (http://creativecommons.org/licenses/by/4.0/).
